# Late-Life Cognition and Dementia in India: New Insights From LASI-DAD

**DOI:** 10.1093/geroni/igaa057.2277

**Published:** 2020-12-16

**Authors:** Jinkook Lee

## Abstract

With more than1.35 billion people, India, the second-most populous country in the world, is soon to experience rapid aging of its population. By2050, India’s older population is projected to reach320 million (about the current size of the entire U.S. population). In this session we introduce the Longitudinal Aging Study in India – Diagnostic Assessment of Dementia (LASI-DAD), a new cohort study designed to advance dementia research to better understand late-life cognition, cognitive aging, cognitive impairment, and dementia, as well as their risk and protective factors. LASI is a prospective, multi-purpose population survey of older adults aged45 and older, representative of the entire country and of each state (N~72,000). LASI-DAD is an in-depth study of late-life cognition and dementia, drawing a sub-sample of older adults aged60 and older from LASI (N~4,300). It administered the Harmonized Cognitive Assessment Protocol (HCAP), which consists of a pair of in-person interviews, one with the target respondent and one with an informant nominated by the respondent. The respondent interview includes a neuropsychological test battery designed to measure a range of key cognitive domains affected by cognitive aging and Alzheimer’s Diseases. We organize the session to showcase LASI-DAD. Specifically, the session consists of four papers, including: (1) the introduction of the design and methodology, (2) the latent structure of neuropsychological test results, (3) the investigation of the relationship between visual impairment and cognition, and (4) the examination of female disadvantage in dementia and its association with cross-state variations in gender inequality.

